# The relative effects of climatic drivers and phenotypic integration on phenotypic plasticity of a globally invasive plant

**DOI:** 10.3389/fpls.2024.1473456

**Published:** 2024-11-25

**Authors:** Xincong Chen, Jiayu Wang, Wenwen Liu, Yihui Zhang

**Affiliations:** Key Laboratory of the Ministry of Education for Coastal and Wetland Ecosystems, College of the Environment and Ecology, Xiamen University, Xiamen, Fujian, China

**Keywords:** biological invasion, common garden, constraints to plasticity, latitudinal gradient, *Spartina alterniflora*, traits plasticity

## Abstract

**Introduction:**

Understanding the constraints of phenotypic plasticity can provide insights into the factors that limit or influence the capacity of an organism to respond to changing environments. However, the relative effects of external and internal factors on phenotypic plasticity remain largely unexplored. Phenotypic integration, the pattern of correlations among traits, is recognized as an important internal constraint to plasticity. Phenotypic plasticity is critical in facilitating the acclimation of invasive species to the diverse environments within their introduced ranges. Consequently, these species serve as ideal models for investigating phenotypic plasticity and its underlying determinants.

**Methods:**

Here, we collected seeds of a global salt marsh invader *Spartina alterniflora* from seven invasive populations covering the entire latitudinal range in China. These populations were cultivated in two common gardens located at the southern and northern range margins, respectively. We quantified plasticity and variation therein for plant height, shoot density, first flowering day and inflorescence biomass (on a per capita basis). These traits have direct or indirect effects on invasiveness. We examined the relationships between traits plasticity with climatic conditions at site of origin (external factor) and phenotypic integration (internal factor).

**Results:**

We found that plasticity differed according to the trait being measured, and was higher for a trait affecting fitness. Phenotypic variance increased with latitude and temperature at the site of origin was the primary factor affecting phenotypic variation. These results indicated that external abiotic factors directly affected the selection on phenotypic plasticity of *S. alterniflora*.

**Discussion:**

Our study provides a unique viewpoint on assessing the importance of identifying influential factors and mechanisms underlying phenotypic plasticity. Understanding these factors and mechanisms is a critical indicator for invasive and other cosmopolitan species’ responses, establishment, persistence, and distribution under climate change.

## Introduction

Phenotypic plasticity, the ability of a single genotype to produce varying phenotypes, has long been acknowledged as an adaptive strategy for dealing with diverse environmental conditions (Nicotra et al., 2010). Variable plasticity among species or populations indicates divergent selective forces and constraints on maximizing plasticity (Valladares et al., 2007). Given the importance of phenotypic plasticity in species’ response to change of environment, it is critical to identify possible constraints to the trait plasticity. Both external (extrinsically ecological limits such as the abiotic and biotic factors) and internal (intrinsic characteristic of organisms such as the phenotypic integration, genetic cost and ontogeny) factors may limit phenotypic plasticity (van Kleunen and Fischer, 2005; Valladares et al., 2007), and numerous studies have been devoted to explore how these factors affect the magnitude of plasticity (Gianoli and Palacio-López, 2009; Matesanz et al., 2010; Molina-Montenegro and Naya, 2012). To the best of our knowledge, however, there is a paucity of work testing the relative effects of external and internal factors on the plastic response.

The climatic variability hypothesis (CVH) predicts that organisms originating from higher latitudes are likely to exhibit greater phenotypic plasticity (Janzen, 1967; Stevens, 1989). As the extent of climatic variability expands as latitude increases, consequently, populations residing in higher latitudes possess stronger acclimation capabilities to adapt to ever-changing environments (Pither, 2003). Generally, the climatic conditions could be the important external factor to drive phenotypic plasticity of latitudinal populations (Molina-Montenegro and Naya, 2012). However, experimental evidence on the relationships between phenotypic plasticity with specific temperatures at sites of origin was to date lacking (Gianoli, 2004; Ren et al., 2020). Aggressive invaders frequently exhibit broad climatic tolerance and disperse rapidly (Willis and Hulme, 2002). Phenotypic plasticity has been a focus of many studies on invasive plants (Richards et al., 2006; Spector and Putz, 2006; Messier et al., 2017), and several of these studies also found support for the CVH (Molina-Montenegro and Naya, 2012; Li et al., 2016). Studies exploring the relationship between climatic conditions and plasticity thus can promote our understanding of selective regime for plastic responses of invasive species and their dispersion dynamic under climate change.

Phenotypic integration refers to the interconnectedness of traits related to plant growth, reproduction, and fitness. This concept involves correlations related to development or heredity (Pigliucci, 2003; Matesanz et al., 2021), and is recognized as an important internal constraint to plasticity (Valladares et al., 2007). Moreover, phenotypic integration affects the coordination of traits thereby the acclimation of organisms in response to environmental conditions. High phenotypic integration means that there is an increased number of significant associations between a specific trait and other traits within a given environment. Conversely, low phenotypic integration is characterized by fewer significant correlations (Matesanz et al., 2021). If the ratio between the number of significant associations and all possible pairwise trait combinations within a given environment exceeds 20%, it is considered high phenotypic integration (Zimmermann et al., 2016). It seems a general pattern that phenotypic integration would limit the expression of trait plasticity, as more linkage with other traits may constrain the capacity of one trait to change (Schlichting, 1989). However, emerging studies challenge the notion that phenotypic integration could constrain plasticity, they found greater trait variation for each of the associated traits when there is greater phenotypic trait integration. Both phenotypic plasticity and integration can improve the fitness, therefore, they may work synergistically in the acclimation of plant species (Zimmermann et al., 2016; Pireda et al., 2019; Matesanz et al., 2021). These mixed results indicate that phenotypic integration appears to influence trait plasticity in a manner that is environment- or species-dependent. Ideally, studies that cover the entire distribution range of the species may be able to disentangle whether plasticity is mediated by environmental or genetic variation (Hulme, 2008).

Exotic species are often subjected to strong selection from environment in the invaded ranges (Bossdorf et al., 2005). Phenotypic plasticity could improve population fitness in different environments (Barrett, 2015), which suggests that the ability to acclimate to their environment of invasive species may be highly dependent on phenotypic variation (Richards et al., 2006; Godoy et al., 2012). During biological invasion, the variation and evolution of phenotypic plasticity can be studied within a relatively short time scale (Hulme, 2008). Invasive species are therefore good model systems to explore the plastic responses and the factors that influence the magnitude of phenotypic plasticity under changing environments. *Spartina alterniflora* Loisel. (synonym of *Sporobolus alterniflorus* Loisel.) (Peterson et al., 2014) is native to the eastern coasts of the Americas, and is invasive in many other parts of the world. Its occurrence along almost the entire coast of China (from 19°N to 39°N) makes it ideally suited to studies on phenotypic plasticity. Plasticity is vital for *S. alterniflora*, and field surveys and common garden experiments revealed considerable phenotypic variation with latitude for multiple phenotypes (Liu et al., 2017; Chen et al., 2021). Additionally, a common garden experiment found that latitudinal clines in reproductive traits of *S. alterniflora* were maintained over three years, but that such clines in vegetative traits disappeared since the second year (Liu et al., 2020b). These results suggested that some of the clines have a genetic underpinning and others result from epigenetic mechanisms or other maternal carry-over effects. However, there are few accurate quantifications of traits plasticity and their integration in *S. alterniflora*, and the impacts of external and internal factors on phenotypic plasticity have not been explored.

**Figure 1:**
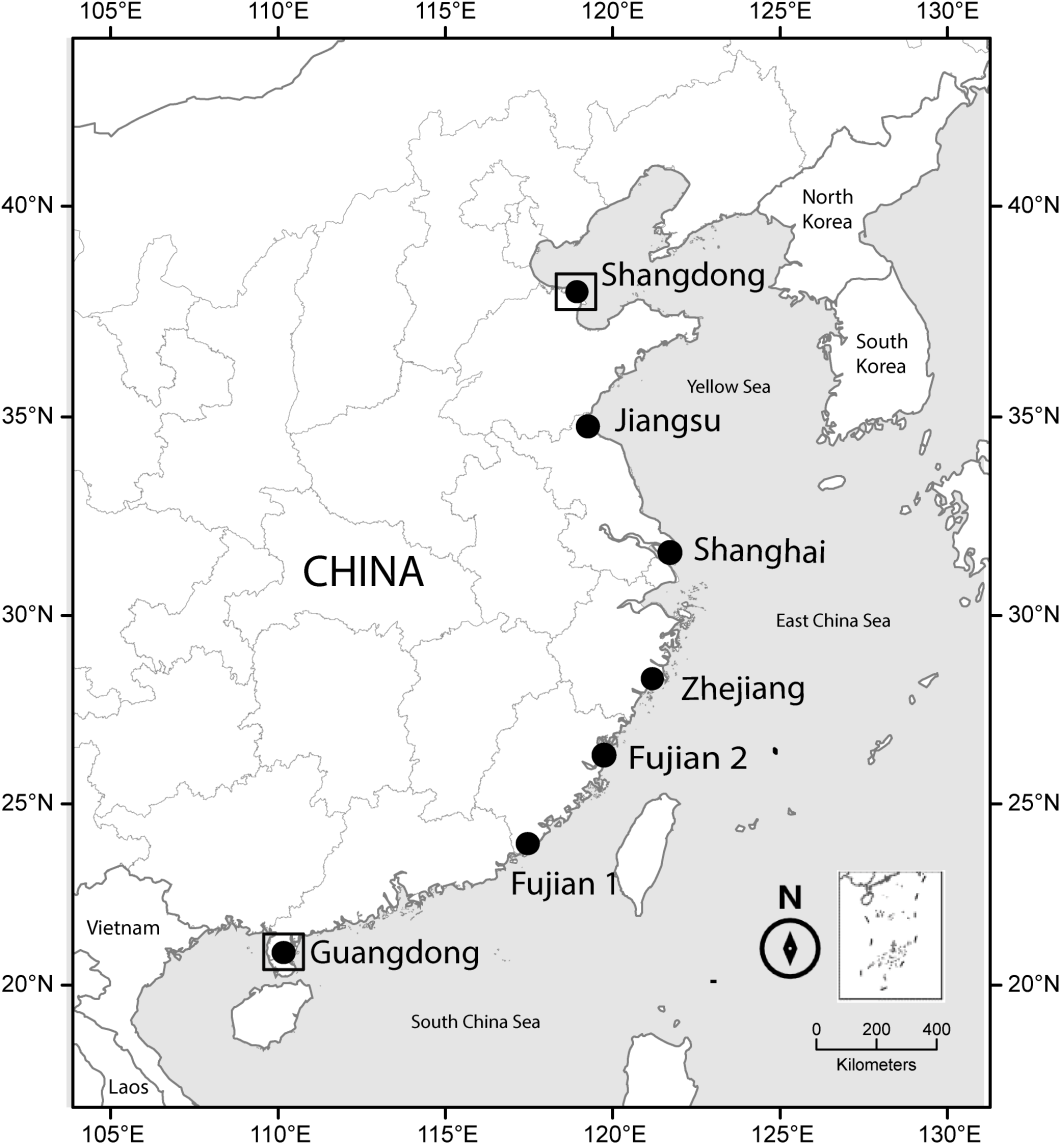
Map of study sites. The inset map, positioned adjacent to the north arrow in the bottom right corner, displays the People’s Republic of China’s Nine-Dash Line in the South China Sea. Sites of population seed sampling are marked with solid circles and sites of common garden are marked with squares.

In this study, we employed a common gardens experiment, which is an effective tool to quantify and understand phenotypic plasticity (Bossdorf et al., 2005). Using genotypes from different locations along an environmental gradient, different clinal patterns in individual traits across common gardens can reveal genotype-by-environment interactions and the effect of local environment conditions on phenotypic plasticity (Cooper et al., 2019). Moreover, multiple common gardens allow us to test the effect of phenotypic integration on plasticity across distinct environments. Seven *S. alterniflora* populations were collected along the entire latitudinal range of the species in China. These populations were grown in two common gardens, one at the southern and one at the northern range margin with distinct difference in climatic conditions (**Figure 1**). We measured four traits, flowering time is a key switch from vegetative growth to sexual reproduction and strongly correlated with generation time (Fenner, 1998), and inflorescence biomass is directly related to the fitness of invaders (Qiao et al., 2019). Together with plant height and shoot density—measures of plant growth architecture—these traits provide responses of the multiple plant organs to changing environments (Violle et al., 2007) and are likely to have direct and indirect effects on competition and distribution of plant invaders (Messier et al., 2017; Qiao et al., 2019). Meanwhile, we gathered data on five climatic factors from the original sites. We addressed these questions: (1) Do the measured traits show different plastic responses between common gardens? (2) Are there latitudinal clines in traits plasticity, and if so, how do climatic conditions drive the variation in phenotypic plasticity along latitude? (3) What are the relative effects of climatic conditions and phenotypic integration on phenotypic plasticity? We hypothesize that (1) inflorescence biomass has higher plasticity than the other traits due to its direct impact on plant fitness; (2) populations from higher latitudes have higher phenotypic plasticity than the other populations. Fluctuating climatic conditions contribute to greater plasticity at high latitudes; (3) climatic conditions affect phenotypic plasticity more than phenotypic integration. The strong selective pressure from abiotic factors along latitude may surpass the variable effect of phenotypic integration on plasticity.

## Materials and methods

### Study species and geographic sampling

*Spartina alterniflora* Loisel., also referred to as smooth cordgrass, is a long-lived grass that can grow between 1 and 3 meters tall. It can flower annually with up to one inflorescence per stem (Chen et al., 2021). Highly viable seeds enable *S. alterniflora* to spread over long distances with ocean currents (i.e., sexual reproduction), and vigorous rhizomes contribute to local expansion (i.e., clonal growth) (Daehler and Strong, 1994). *Spartina alterniflora* originates from the Atlantic and Gulf coasts along the eastern coasts of the Americas but has evolved into a highly problematic invader worldwide (Strong and Ayres, 2013). In 1979, *S. alterniflora* was brought into China for the first time and has spread rapidly (Xu and Zhuo, 1985). Now, *S. alterniflora* is found along the entire coast of China, making it the largest area invaded by a species in the world (Zhang et al., 2017).

In 2018, we collected seeds from seven *S. alterniflora* populations from September to October (end of growing season) at different geographic sites from 21°N to 38°N, covering the entire distribution of coastal China (**Figure 1**; **Supplementary Table S1**). Because *S. alterniflora* populations in China resulted from a single genetic admixture occurrence which resulted in multiple novel recombinant genotypes (Qiao et al., 2019), each population was treated as a single geographic genotype. At each site, we selected two subsites, separated by 2-3 km, and gathered seeds from five 0.5 m × 0.5 m quadrats at each subsite. The quadrats were spaced at least 30 meters apart and derived from distinct clones, thus treating the seeds gathered from each quadrat as separate seed families. In total, we collected mature, whole inflorescences from 70 quadrats. Although seeds from the same plant may only be half-sibs, such collections are a well-established method for examining the impact of genetics on traits. We individually placed the filled seeds from each quadrat into sealed plastic bags containing 10 PSU seawater and stored them at 4°C.

### Common gardens experiment

To assess plasticity and integration of functional traits of *S. alterniflora*, two common gardens were built at the *S. alterniflora* distribution margins in China. They were located at Guangdong (GD, low latitude, 21.12°N, 110.31°E) and Shandong (SD, high latitude, 37.69°N, 118.84°E), respectively, in which we cultivated in parallel plants of the same seed families collected in the seven populations of *S. alterniflora* (**Figure 1**; **Supplementary Table S1**). Precipitation may not be the most important abiotic factor influencing plant growth and distribution in regularly inundated coastal wetland (Pennings and Bertness, 2001). Additionally, the temperature could be the most critical factor to *S. alterniflora* growth (Liu et al., 2020a), development (Chen et al., 2021), and reproduction (Liu et al., 2020b), because temperature has direct effects on physiological function (Pau et al., 2011). Therefore, we particularly wanted to focus on the effects of differences in temperature between gardens, and minimized differences in other environmental conditions, such as precipitation, herbivory, and edaphic condition. To shield the plants from rain, we covered the top of the common gardens with transparent plastic film to shield the rain. To keep out insect pests, we also covered the lower parts of the sides with a 1-m high plastic film and the remaining parts with insect-proof gauze. The latter allowed for sufficient air circulation to keep the ambient temperature consistent with the temperature of the surrounding environment. We initiated the germination of seeds from every family in March 2019, the seeds were nurtured in a greenhouse at Xiamen in Fujian (24°N) until they reached a height of 7-8 cm. Subsequently, we transferred the seedlings to their respective garden locations. Each common garden was equipped with 10 individual rectangular plastic pools (dimensions: 1.1 m in length, 0.8 m in width, and 0.3 m in depth). Inside each pool, there were 7 plastic pots (dimensions: 18 cm in diameter, 24 cm in height) filled with a mixture consisting of 50% Jiffy’s peat substrate (Jiffy Products International BV, Moerdijk, Netherlands) and 50% vermiculite by volume. Two seedlings from each of the ten seed families of each of the seven populations was randomly assigned to two plastic pots in each pool (2 seedlings × 10 seed families × 7 populations × 2 gardens = 280 seedlings). The pools were filled with water that we adjusted to 10 practical salinity units (PSU) by dissolving sea salt into it, and the water level in the pots was maintained at the same height as the soil level. Fresh water was added to the pools every second day, and the salt water was entirely replaced monthly to keep the salinity stable. Above conditions were within the range experienced for salt marshes invaded by *S. alterniflora*, although it does not completely mimic the conditions in nature, the continuously waterlogged soil corresponds to what the plants experience in nature due to regular flooding. In October 2019 (end of growing season), we measured the plants (see the section ‘Trait measurements’).

### Traits measurement

We measured four traits that relate to performance and fitness of *S. alterniflora*: two vegetative growth traits (plant height and shoot density), one key phenological trait (days until flowering), and one reproductive trait (inflorescence biomass). To record the date on which the first shoot in each pot began flowering, we checked the pots every four days during the entire growing season (April to October), and we calculated the first flowering day represented by the days elapsed since January 1st (Chen et al., 2023). A shoot was considered flowering once the inflorescence had protruded from the topmost leaf and visible pollen was present (Chen et al., 2021). In October, in each pot, we tallied the shoots that were taller than 25 cm and calculated shoot density by dividing the shoot count by the area of the pot. There are often many very short shoots in *S. alterniflora* stands, yet their contribution to overall standing biomass is negligible (Morris and Haskin, 1990; Liu et al., 2020b). These shoots were almost never able to complete their life history in this study. In each pot, the height of the three tallest shoots was measured. The inflorescences of these shoots were then collected, dried at 60°C until their mass remained constant, and weighed (Liu et al., 2020b). Therefore, the inflorescence biomass was on a per capita basis, i.e., averaged across three inflorescences from the tallest stems. The traits data of two pots from the same seed family were averaged before statistical analyses.

### Climate variables

Local environmental conditions at the original sites could have driven the variation in phenotypic plasticity. Therefore, we obtained long-term climatic data (including MAT, AGDD, MCDT, MWDT and TAR as listed in **Supplementary Table S1**) for every sampled site covering the years from 1981 to 2020, which were sourced from the China Meteorological Data Service Center (CMDC, http://data.cma.cn) (Chen et al., 2023). This duration covers nearly all of the years that *S. alterniflora* spread vigorously and occupied almost the entire Chinese coastline following its introduction in 1979. We calculated Pearson coefficients for every pairwise combination of the climatic variables to determine collinearity among them, and to reduce the collinearity among these variables, we performed the principal component analysis (PCA) on the climatic variables. Finally, within each common garden, we placed a HOBO temperature logger (MX2202) at a height of one meter above the ground at the garden’s center to record air temperature every 10 minutes throughout the experiment. The recorded temperature data were used to calculate the five variables above (**Supplementary Table S1**) during the experiment for both common gardens.

### Statistical analyses

We assessed normality via Shapiro-Wilk’s test and homogeneity of variance via Levene’s tests. Since results of these tests were not well satisfied, log[x]-transformed data was applied in following analyses when necessary. To test whether inflorescence biomass had higher plasticity than the other traits (hypothesis 1), firstly, we investigated the phenotypic differences of each trait between common gardens and among populations, via using two-way ANOVAs incorporating common gardens and populations and their interaction as fixed effects. Secondly, phenotypic plasticity of each trait was calculated as phenotypic plasticity index (PIv) for each seed family, PIv is calculated as the difference between the maximum and minimum values between gardens, divided by the maximum value; the PIv varies from 0 to 1, where 0 denotes an absence of plasticity and 1 denotes the greatest plasticity (Valladares et al., 2007; Ren et al., 2020). This approach is a robust, simple and widely used index when two environments are considered (Valladares et al., 2006). We then applied one-way ANOVAs followed by Tukey-Kramer HSD *post-hoc* tests to compare the PIv values among the four traits including data from all seven populations. Finally, in order to accounts for population variations in PIv values, we created a mixed effect model for PIv values via R function “lmer” from the “lme4” package (Bates et al., 2015). This model employed trait, population, and interaction of trait and population as fixed factors, and treated seed family nested within each sampling subsite as a random effect.

To test whether phenotypic plasticity was stronger for populations from higher latitudes than other ones (hypothesis 2), firstly, we detected whether there was variation in plasticity among populations, via creating mixed effect models for each measured trait with garden site, latitude of origin, and interaction of garden site and latitude of origin as fixed factors, and subsite and seed family as random effects (due to multiple levels being sampled in a single site). Secondly, we regressed the PIv of each trait on latitude of origin. Further to compare the slopes of these regressions, we performed a linear regression model with PIv as the response variable and interaction between latitude of origin and trait as the predictor variable. Finally, to test the effects of local environmental conditions on traits plasticity (hypothesis 2), we regressed the PIv of each functional trait with climate PC1 at sites of origin.

To test whether climatic conditions contribute more to phenotypic plasticity than phenotypic integration (hypothesis 3), we performed regression analyses with phenotypic plasticity as dependent variable and phenotypic integration as independent variable in both gardens. The degree of phenotypic integration for each trait was assessed by counting the significant correlations (*P* < 0.1; Pearson’s correlation) it had with every other trait. In this study, for each of the seven populations in both gardens, we computed phenotypic integration per trait (n = 4 traits × 7 populations × 2 gardens = 56). Mean values of phenotypic plasticity of each trait for each population were log-transformed before analysis (log[x]) to achieve better normality in error distribution and consistency in variance.

We performed all analyses with R statistical software (R Development Core Team, 2018).

## Results

**Figure 2:**
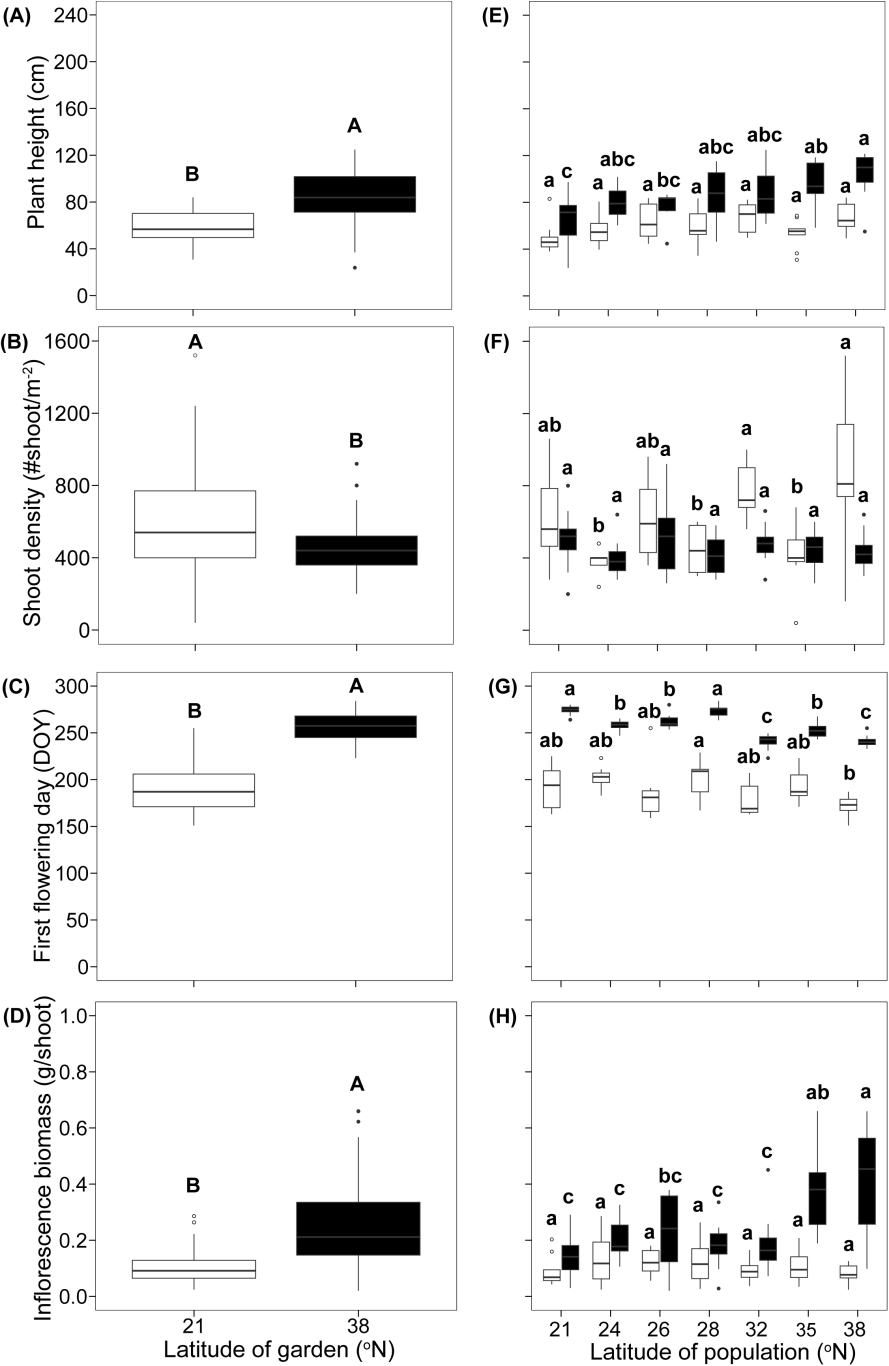
The plant height **(A, E)**, shoot density **(B, F)**, first flowering day **(C, G)**, and inflorescence biomass **(D, H)** in different common gardens and among populations in each common garden. The white boxes represent low latitude garden, and black boxes represent high latitude garden. Different capital letters represent significant difference between gardens **(A–D)**, different lowercase letters represent significant difference among populations **(E–H)**. DOY, day of year.

There were significant differences in the overall averages of the four functional traits between gardens. *Spartina alterniflora* grew 31% smaller (**Figure 2A**; *F*_1,114_ = 71.25, *P* < 0.001), had 29% higher shoot density (**Figure 2B**; *F*_1,115_ = 16.73, *P* < 0.001), flowered 27% earlier (**Figure 2C**; *F*_1,114_ = 740.57, *P* < 0.001), and had 56% lighter inflorescence biomass (**Figure 2D**; *F*_1,114_ = 64.01, *P* < 0.001) in the common garden at low-latitude than in the garden at high-latitude. Besides, these traits were all significantly differed among populations (**Figures 2E–H**).

**Figure 3:**
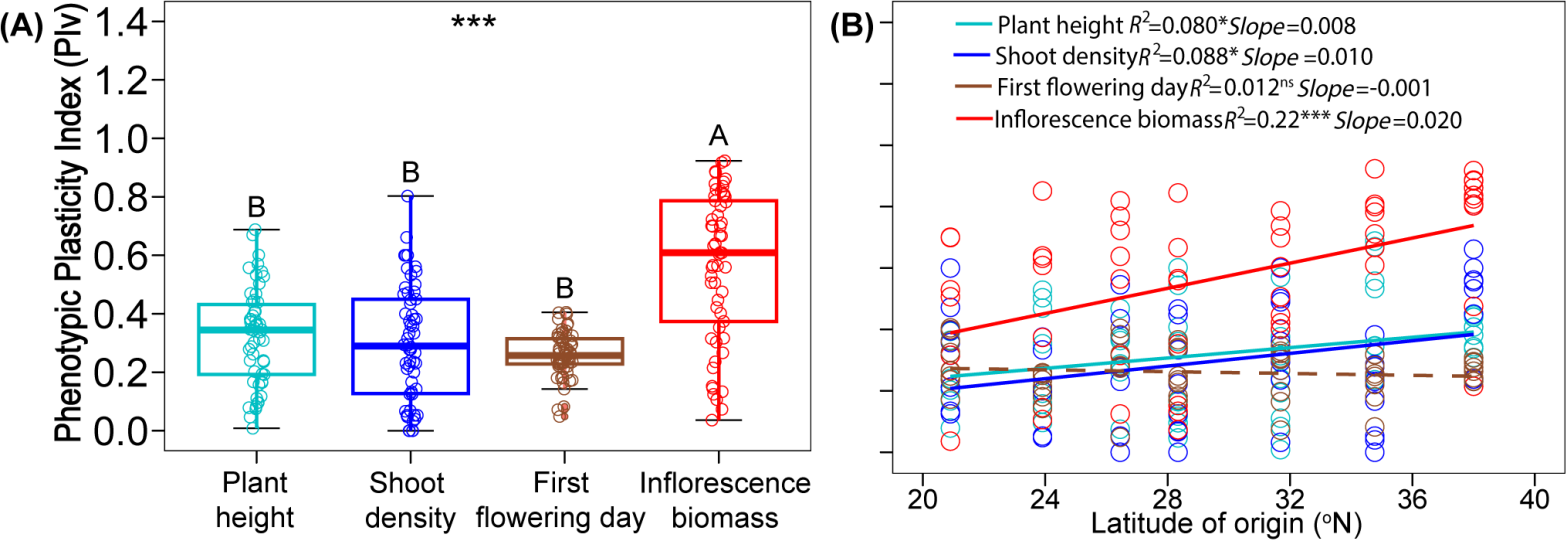
Comparison of Phenotypic Plasticity Index (PIv) among traits **(A)**, significances are shown by different letters. Relationships between PIv of traits with latitude of origin **(B)**, significant relationships are marked with solid lines and non-significant one is marked with dash line. Significance levels: ns, *P* > 0.05; *, *P* < 0.05; ***, *P* < 0.001.

Phenotypic plasticity index (PIv) varied significantly among the four traits (**Figure 3A**; **Supplementary Table S3**; *F*_3, 199_ = 39.56, *P* < 0.001). PIv of inflorescence biomass was 75%, 87%, 115% higher than the PIv of plant height, shoot density, and first flowering day, respectively. These three traits did not differ from one another (**Figure 3A**). There was significant latitudinal variation in the PIv of plant height, shoot density, and inflorescence biomass, with northern populations exhibiting greater plasticity than southern populations (**Figure 3B**; plant height: *F*_1, 52_ = 4.52, *P* = 0.038; shoot density: *F*_1, 56_ = 5.38, *P* = 0.024; inflorescence biomass: *F*_1, 55_ = 15.17, *P* < 0.001). The PIv of the first flowering day did not differ with latitude (**Figure 3B**; *F*_1, 56_ = 0.68, *P* = 0.412). This was also confirmed by the mixed effects models, garden and original latitude had significant interaction for plant height, shoot density, and inflorescence biomass, but not for first flowering day (**Supplementary Table S4**).

**Figure 4:**
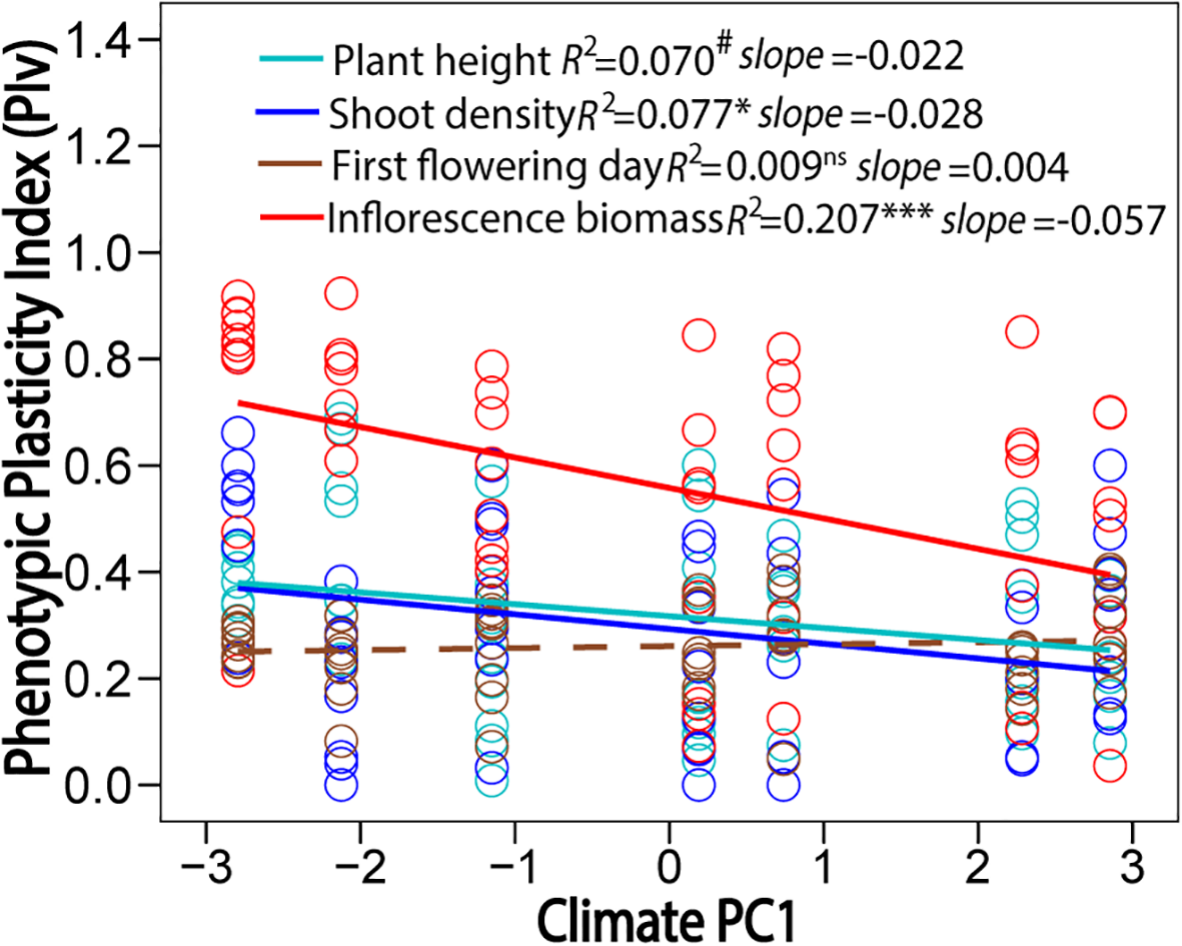
Relationships of phenotypic plasticity index (PIv) of plant height, shoot density, first flowering day, and inflorescence biomass with climate PC1 at sites of origin. Positive PC1 represented high and stable temperatures along latitude. Significant relationships are marked with solid lines and non-significant one is marked with dash line. Significance levels: ns, *P* > 0.06; # marginal, *P* = 0.05-0.06; *, *P* < 0.05; ***, *P* < 0.001.

**Figure 5:**
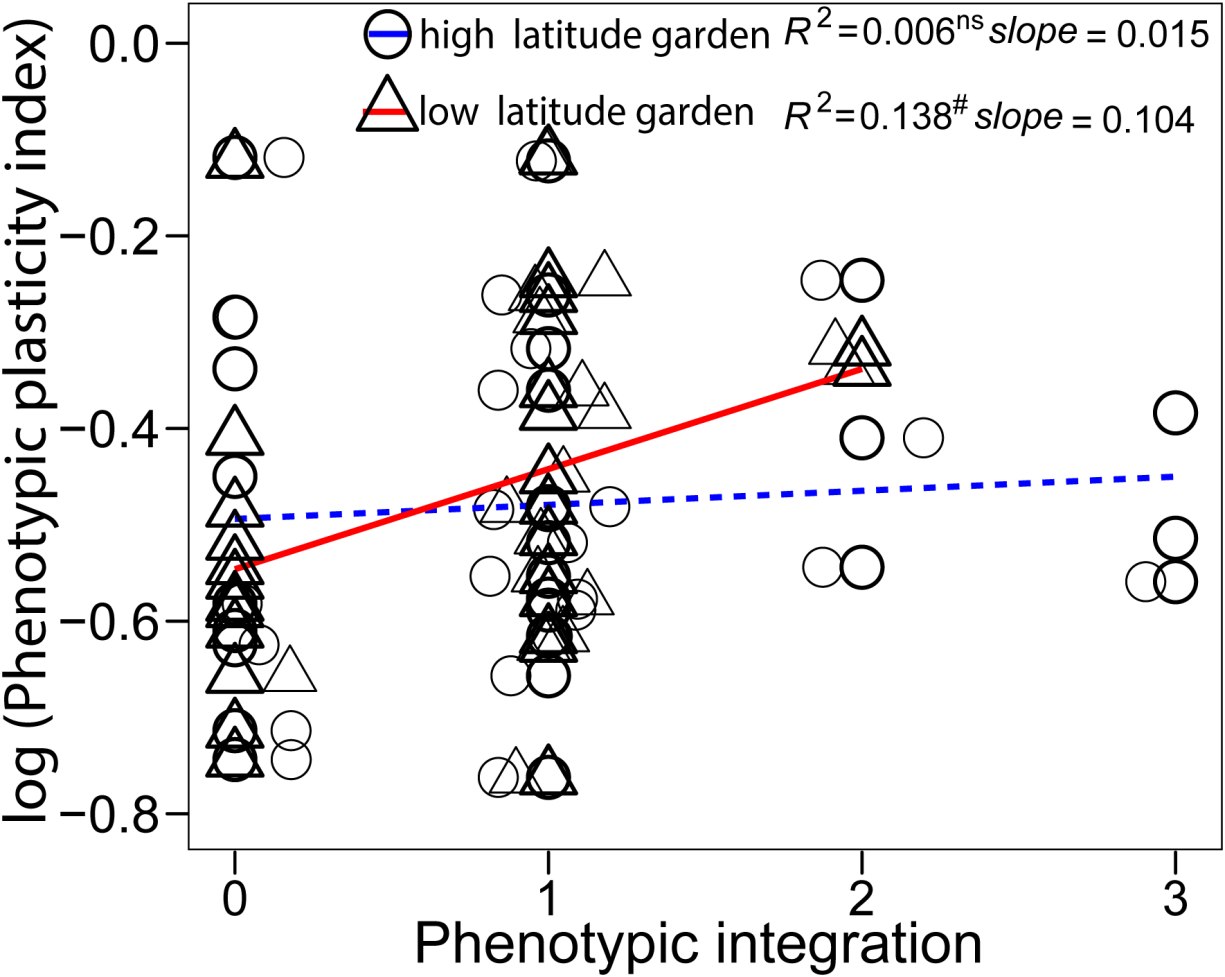
Relationship between trait plasticity and phenotypic integration in both high- and low- latitude garden. Each point represented a single trait, there were 4 traits of 7 populations in each regression analysis. Significant relationship is marked with solid line and non-significant one is marked with dash line. Jitter was used to distribute the data points. Significance levels: ns, *P* > 0.1; # marginal, *P* < 0.1.

The five climatic factors at original sites (**Supplementary Table S1**) were closely related with each other and latitude, with the exception of the MWDT (**Supplementary Table S2**). The MAT, AGDD, and MCDT had negative correlations with latitude, and the TAR had positive correlation latitude (**Supplementary Table S2**). The first two principal components were selected to depict the variation in climate variables along latitude. The PC1 explained ~80% of variation in these climate variables, and positive PC1 represented warmer and more stable climatic conditions (**Supplementary Figure S1**). The climate variables at sites of origin were negatively correlated with the PIv of plant height, shoot density, and inflorescence biomass (**Figure 4**), with lower temperature and greater temperature variation correlating to greater phenotypic plasticity. The PIv of first flowering day was not correlated to any climatic variable (**Figure 4**). Phenotypic plasticity and integration had a marginally significant positive relationship in the low-latitude garden (**Figure 5**; *F*_1, 26_ = 4.16, *P* = 0.052) and non-significant in the high-latitude garden (**Figure 5**; *F*_1, 26_ = 0.16, *P* = 0.688).

## Discussion

We found trait-based differences in phenotypic plasticity, and degree of plasticity increased with latitude. These latitudinal clines suggest that phenotypic plasticity could be considered as a functional trait under selection (Nicotra et al., 2010; Ren et al., 2020). Such variation can be trait specific and is driven by the climatic conditions at the different latitudes. If this variation in phenotypic plasticity enhances fitness, it may have facilitated the successful invasion of *S. alterniflora*. Besides, we found an impact of phenotypic integration on plasticity, it differed by common garden site. External abiotic factors have a larger effect on phenotypic plasticity than phenotypic integration.

### Different plastic responses among traits

Our results showed that all measured traits exhibited considerable phenotypic variation between common gardens. Plasticity differed by trait, and we found the highest plasticity in inflorescence biomass, which serves as an indicator of fitness (Johnson et al., 2010). Higher plasticity in traits that contribute to fitness may increase environmental tolerance and thereby widen the niche breadth (Poorter et al., 2012; Castillo et al., 2014). However, we found relatively low plasticity in the first flowering day. Flowering phenology is quite plastic and usually speeds up at higher temperatures (Menzel et al., 2006; Richardson et al., 2006; Augspurger and Zaya, 2020), surprisingly, its plasticity was low for *S. alterniflora.* Having said this, many studies on variation in phenology under climate warming have shown that it is particularly the spring-flowering species that speed up their phenology, not the autumn-flowering species (like *S. alterniflora*) (Miller-Rushing and Primack, 2008; Alecrim et al., 2023). On the other hand, physiological adaptations to extreme climatic conditions, such as high temperature in the low-latitude garden in the present study may constrain *S. alterniflora* growth (Liu et al., 2020a), thus reduce the need for plasticity in phenological traits (Cooper et al., 2019). Besides, we found plant height and shoot density had similar plasticity as time to flowering.

### Latitudinal variation in phenotypic plasticity

We found phenotypic variance increased with latitude, there were positive relationships between plasticity of plant height, shoot density, and inflorescence biomass with population latitude. These results were consistent with the CVH, which stated that organisms from higher latitudes have greater phenotypic plasticity (Molina-Montenegro and Naya, 2012). Differentiation in plasticity among populations may indicate genetic control of plasticity in invasive *S. alterniflora* to a certain extent. We found significantly stronger evidence for genetic based variation in phenotypic plasticity of the inflorescence biomass than plant height and shoot density. Previous common garden experiments found that reproductive traits of *S. alterniflora* exhibited contrasting latitudinal clines between common gardens, but the plant height or shoot density showed similar clines (Liu et al., 2017; Qiao et al., 2019). These results also indicated stronger latitudinal variation in plasticity of reproductive traits than the vegetative ones. In this study, the significant clinal variation in plasticity of inflorescence biomass indicated a potentially adaptive phenotypic plasticity in this trait. However, this clinal variation in plasticity was not ubiquitous among traits; we found no latitudinal cline in the first flowering day plasticity. Results of mixed models further supported this finding, indicating significant interactions between garden site and population latitude affecting plant height, shoot density, and inflorescence biomass, but not first flowering day. Studies of phenological plasticity have yielded mixed results. For example, Ren et al. (2020) also found no latitudinal cline in the PIv of flowering time, however, Cooper et al. (2019) found that phenological plasticity tended to decrease with increasing latitude. These contrasting results may be due to differences in plant life forms and habitats. In our study, we found no difference in flowering time plasticity across latitudinal populations. Although many studies have suggested the high genetic diversity of *S. alterniflora* populations in China (Wang et al., 2012; Qiao et al., 2019), the genetic differentiation for flowering time plasticity across populations may be not significant, as reported in another *Spartina* species, *Spartina densiflora* (Castillo et al., 2018).

### Effect of climatic conditions on phenotypic plasticity

Temperature at the site of origin was the primary factor affecting phenotypic variation. *Spartina alterniflora*, in the United States (native range), occurs at higher latitudes than in China (invasive range) (Liu et al., 2020b). In China, however, *S. alterniflora* experienced greater environmental fluctuations at high latitudes than the native range (higher TAR) (Liu et al., 2020a; Chen et al., 2023). We found that greater fluctuation in temperature (higher TAR) at higher latitudes was indeed associated with greater plasticity in plant height, shoot density, and inflorescence biomass of northern populations in invasive China. The northern populations with higher phenotypic plasticity exhibited higher overall performance compared to those with lower plasticity. Phenotypic plasticity is often regarded as highly beneficial for plants (Baker, 1974), as it is believed to enhance environmental adaptability, or fitness homeostasis (Valladares et al., 2014). This suggests that phenotypic plasticity of *S. alterniflora* is a potentially adaptive response to novel environmental conditions in the invasive range. Additionally, lower temperature (i.e., mean annual temperature, growing degree days, coldest daily temperature, and warmest daily temperature) may also relate to higher plasticity of northern populations, because populations that experience predictable winter freezing may show greater plasticity (Cooper et al., 2019). Although *S. alterniflora* occupies lower latitudes in the invasive range than in the native range, the lower latitude portions of both ranges have similar ambient temperatures (Liu et al., 2020a). Climatic conditions in these low latitudes exceed the optimum temperature for vegetative growth and may limit the aboveground activity of *S. alterniflora* (Liu et al., 2020a). Therefore, reduced plasticity in southern *S. alterniflora* populations may be due to physiological constraints related to poor heat tolerance, as stress can limit plant phenotypic plasticity because of higher cost (Stotz et al., 2021). We found no relationships between plasticity of the first flowering day and climatic variables in *S. alterniflora*, indicating no local adaptation, as a previous study has found parallel latitudinal clines in first flowering day of this species in different common gardens (Chen et al., 2023). Such patterns suggest that different genotypes of *S. alterniflora* maintain similar degree of phenotypic response to environmental variation. This variation in plasticity should be subject to selection.

Understanding local adaptation to past long-term climatic condition allows us to contextualize contemporary ecological variation and predict distribution dynamics under changing environments (Woods et al., 2012). Latitude represents a proxy for multiple variables, and plastic responses of plants have been documented with respect to many abiotic factors, including temperature, precipitation, and soil (Dudley, 2004). Temperature is a key factor in affecting plastic adaptation along clines (Atkin et al., 2006) and has received particular interest in the context of global warming. Since our results suggested that both low and fluctuating temperatures favor high plasticity, therefore, when under a continuous and steady warming in the future, the advantages of high plasticity in northern *S. alterniflora* populations may be reduced (Norberg et al., 2012).

### Effect of phenotypic integration on phenotypic plasticity

Phenotypic integration plays a crucial role by enabling organisms to coordinate their traits or by affecting a single trait under given environmental conditions, thereby enhancing their acclimation ability. The four traits that measured in this study represented the key growth, development, and reproduction in plant life history, can provide a comprehensive representation of phenotypic integration in plants. Although many studies used more than five traits to calculate phenotypic integration (Pigliucci and Marlow, 2001; Zimmermann et al., 2016; Matesanz et al., 2021), they also categorized the traits into growth, development, reproduction, and so on. There was a weak effect of phenotypic integration on phenotypic plasticity of *S. alterniflora* in this study. Changes in environmental conditions can affect both the trait values and trait-trait correlations (Wood and Brodie, 2015). We found no substantial association between phenotypic integration and plasticity in both common gardens. Only a marginally positive relationship between integration and plasticity in the low-latitude garden. High temperature in the common garden at low-latitude could be stressful for *S. alterniflora*, such positive effect could be essential for plants to deal with stressful environment. Greater phenotypic traits integration contributed to higher level of trait plasticity, thereby enhancing the flexibility and acclimation of organisms in response to environmental conditions (Ghalambor et al., 2007; Zimmermann et al., 2016; Matesanz et al., 2021). A previous study has suggested that the effects of internal constraints to plasticity depend on environmental conditions, with stronger effect within stressful environments. Such phenomenon may be due to the physiological limits on plants growth (Steinger et al., 2003). Besides, phenotypic variation in traits within a given environment may also diminish the effect of phenotypic integration on plasticity (Matesanz et al., 2021). Our results suggest that phenotypic integration does not universally influence trait plasticity. Nevertheless, the determinants and mechanisms underlying phenotypic plasticity are complex and far from resolved (DeWitt et al., 1998). Further empirical or theoretical evidences are needed to clarify the specific role of integration as a potential constraint on plasticity (Valladares et al., 2007).

## Conclusion

Our results indicated that phenotypic integration did not affect plasticity of *S. alterniflora* to a large extent, and its plasticity is more driven by external environments (e.g., the temperatures in this study). This scenario potentially implies that plants exhibit substantial plasticity across environments. Simultaneously, phenotypic integration may play a comparatively modest role in regulating overall traits, thereby enabling individual traits to independently respond to changing environments. In our study, the modest effect of phenotypic integration on trait plasticity may be attributed to the few plant traits were employed. Further research is needed to include more traits to thoroughly elucidate the intricate interplay between genetic, developmental, ecological, and evolutionary influences on the extent and limits of phenotypic plasticity. By unraveling these complex relationships, we can gain a deeper understanding of how organisms navigate and adapt to their changing environments. In any case, our study provides a novel view to estimate the constraints of phenotypic plasticity. It is a critical indicator for the species’ responses, establishment, persistence, and distribution under climate change.

## Data Availability

The raw data supporting the conclusions of this article will be made available by the authors, without undue reservation.
