# Supplementary material for: The relative effects of climatic drivers and phenotypic integration on phenotypic plasticity of a globally invasive plant
Source: Front Plant Sci. 2024 Nov 25;15:1473456. doi: 10.3389/fpls.2024.1473456 (PMC11625578; doi:10.3389/fpls.2024.1473456)
Supplement: Supplementary file 1 [file DataSheet1.docx]

Supplementary Materials

**Appendix S1**

**Supplementary Tables**

**Table S1.** Climatic data from locations of common garden experiments (C) and original sampling locations (P). Mean annual temperature (MAT), annual growing degree days (AGDD), mean coldest daily temperature (MCDT), mean warmest daily temperature (MWDT), and temperature annual range (TAR, interval between MCDT and MWDT) data are from long-term averages (1981-2020) for sampling locations and from 2019 in each common garden. Locations are ordered from low to high latitude.

| Experiment | Locations/  Abbreviations | Latitude | Longitude | MAT  (°C) | AGDD  (≥ 10°C) | MCDT  (°C) | MWDT  (°C) | TAR |
| --- | --- | --- | --- | --- | --- | --- | --- | --- |
| C | Guangdong  (GD) | N21 | E110 | 28.0 | 10011.1 | 11.8 | 38.0 | 26.2 |
| P |  |  |  | 23.6 | 8621.7 | 9.0 | 32.4 | 23.4 |
| P | Fujian 1 (FJ 1) | N24 | E117 | 21.5 | 7771.8 | 7.1 | 31.1 | 24.0 |
| P | Fujian 2 (FJ 2) | N26 | E120 | 19.5 | 6920.0 | 3.8 | 33.0 | 29.2 |
| P | Zhejiang (ZJ) | N28 | E121 | 17.9 | 5980.8 | 1.0 | 31.2 | 30.3 |
| P | Shanghai (SH) | N32 | E122 | 17.0 | 5554.7 | -2.2 | 33.7 | 35.9 |
| P | Jiangsu (JS) | N35 | E119 | 14.4 | 4772.2 | -7.1 | 32.0 | 39.1 |
| P | Shandong (SD) | N38 | E119 | 13.6 | 4695.4 | -9.9 | 32.5 | 42.4 |
| C |  |  |  | 17.1 | 5890.7 | -3.3 | 37.3 | 40.6 |

**Table S2.** Pearson’s correlation coefficients (r) for all pairwise relationships between latitude and five climate variables (long-term averages); mean annual temperature (MAT), annual growing degree days (AGDD), mean coldest daily temperature (MCDT), mean warmest daily temperature (MWDT), and temperature annual range (TAR) at sites of origin for each population. Entries in bold indicate statistically significant results. Significance levels: ***P* < 0.01, ****P* < 0.001.

| Factors | Latitude | MAT | AGDD | MCDT | MWDT | TAR | |
| --- | --- | --- | --- | --- | --- | --- | --- |
| Latitude | 1.00 |  |  |  |  |  |  |
| MAT | **-0.99***** | 1.00 |  |  |  |  |  |
| AGDD | **-0.97***** | **0.99***** | 1.00 |  |  |  |  |
| MCDT | **-0.99***** | **0.99***** | **0.97***** | 1.00 |  |  |  |
| MWDT | 0.24 | -0.18 | -0.20 | -0.23 | 1.00 |  |  |
| TAR | **0.99***** | **-0.97***** | **-0.96**** | **-0.99***** | 0.34 | 1.00 |  |

**Table S3.** ANCOVA (mixed models) results of Phenotypic Plasticity Index (PIv) with trait, population, and their interaction as fixed factors, and seed family nested within each sampling subsite as random effect. Entries in bold indicate statistically significantly results (*P* < 0.05).

| Source | *d.f.* | *F* | *P value* |
| --- | --- | --- | --- |
| Trait | 3, 199.00 | 39.56 | **< 0.001** |
| Population | 6, 199.00 | 5.68 | **< 0.001** |
| Trait × Population | 18, 199.00 | 2.47 | **0.001** |

**Table S4.** ANCOVA (mixed models) results of each plant trait with garden site, latitude, and their interaction as fixed factors, and subsite and seed family as random effects. Entries in bold indicate statistically significantly results (*P* < 0.05).

| Traits | Garden site | | | Latitude | | | Garden site * Latitude | | |
| --- | --- | --- | --- | --- | --- | --- | --- | --- | --- |
|  | *d.f.* | *F* | *P value* | *d.f.* | *F* | *P value* | *d.f.* | *F* | *P value* |
| Plant height | 1, 190.18 | 0.90 | 0.344 | 1, 190.68 | 28.06 | **< 0.001** | 1, 189.86 | 8.12 | **0.005** |
| Shoot density | 1, 183.26 | 3.28 | 0.072 | 1, 11.97 | 1.45 | 0.252 | 1, 182.67 | 7.90 | **0.005** |
| First flowering day | 1, 181.61 | 42.11 | **< 0.001** | 1, 12.16 | 11.66 | **0.005** | 1, 181.12 | 1.41 | 0.236 |
| Inflorescence biomass | 1, 188.01 | 10.83 | **0.001** | 1, 12.06 | 10.20 | **0.008** | 1, 187.40 | 25.10 | **< 0.001** |

**Supplementary Figure**


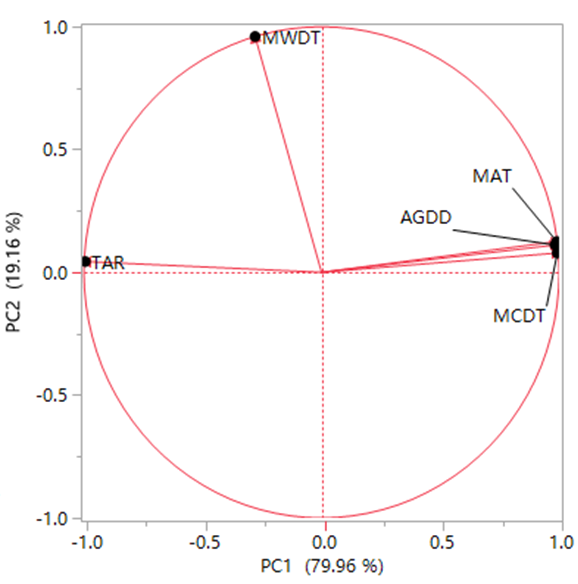


**Figure S1.** Principal components analysis (PCA) of climate variables at sites of population origins. The climate variables include annual growing degree days (AGDD), mean annual temperature (MAT), mean coldest daily temperature (MCDT), mean warmest daily temperature (MWDT), and temperature annual range (TAR, interval between MCDT and MWDT).
